# Region-Based Static Video Stitching for Reduction of Parallax Distortion

**DOI:** 10.3390/s21124020

**Published:** 2021-06-10

**Authors:** Keon-woo Park, Yoo-Jeong Shim, Myeong-jin Lee

**Affiliations:** 1 The Information Technology & Mobile Communications Biz., Samsung Electronics, Suwon-si 16677, Gyeonggi-do, Korea; kw92.park@samsung.com; 2 School of Electronics and Information Engineering, Korea Aerospace University, Goyang-si 10540, Gyeonggi-do, Korea; yoojeong2018@kau.kr; 3 Deptartment of Smart Drone Convergence, Korea Aerospace University, Goyang-si 10540, Gyeonggi-do, Korea

**Keywords:** video stitching, homography estimation, semantic segmentation, region-based video stitching

## Abstract

In this paper, we propose a semantic segmentation-based static video stitching method to reduce parallax and misalignment distortion for sports stadium scenes with dynamic foreground objects. First, video frame pairs for stitching are divided into segments of different classes through semantic segmentation. Region-based stitching is performed on matched segment pairs, assuming that segments of the same semantic class are on the same plane. Second, to prevent degradation of the stitching quality of plain or noisy videos, the homography for each matched segment pair is estimated using the temporally consistent feature points. Finally, the stitched video frame is synthesized by stacking the stitched matched segment pairs and the foreground segments to the reference frame plane by descending order of the area. The performance of the proposed method is evaluated by comparing the subjective quality, geometric distortion, and pixel distortion of video sequences stitched using the proposed and conventional methods. The proposed method is shown to reduce parallax and misalignment distortion in segments with plain texture or large parallax, and significantly improve geometric distortion and pixel distortion compared to conventional methods.

## 1. Introduction

With the development of information and communications technology (ICT) such as 5G and artificial intelligence and changes to the content creation environment, there is a growing demand for immersive media [1,2,3,4], which refers to a medium that conveys information of all types of senses in the scene to maximize immersion and presence for user satisfaction. Immersive media may include multisensory information such as high-quality visual information, multichannel audio information, and tactile information. In particular, as high-quality visual information, virtual reality (VR) media has attracted much attention because it can maximize immersion of users in 3D or ultra high definition (UHD) media. VR media are applied in many fields, such as broadcasting, education, and games, and they are being developed or planned by many companies as a core application service in the 5G era with augmented reality (AR) media [5].

As VR media change from graphic to real images, various 360-degree image capturing equipment types and shooting techniques are being developed. A 360-degree image is synthesized using images taken using a plurality of cameras with wide-angle or fisheye lenses. Generally, 360-degree cameras are configured to shoot images from all directions by radially arranging the cameras of a narrow field of view (FoV) around the same point, and then stitching the captured images offline.

A stitching process is required to generate a 360-degree or panoramic photo-realistic image from images captured by a plurality of cameras [6,7,8,9]. Estimation of the correct alignment to relate various pairs of images, a choice of a final compositing surface to warp aligned images, and seamless cutting and blending of overlapped images are required for image stitching even in the presence of parallax, lens distortion, scene motion, and exposure difference. The stitching process is a critical step in determining the quality of panoramic images, and research is being actively conducted to improve stitching performance [8,9,10,11,12,13].

Three hundred and sixty-degree image stitching tools use the intrinsic and extrinsic parameters of the cameras used in the shooting to stitch images. Commercial 360-degree imaging equipment selects intrinsic parameters according to the camera model used and extrinsic parameters specific to the rig of a specific structure used to mount cameras. The structure of commercial 360-degree video recording equipment is generally set so that the optical axes of the cameras pass through the same point, and the cameras photograph radially using an omni-directional angle of view. Thus, only the rotational transform component is present in the extrinsic parameters of the cameras; the translational component is absent or small enough to be ignored. In such a setting, parallax distortion in a wide-angle panorama or a 360-degree stitched image rarely occurs.

However, when the optical axes of the cameras are not in a radial arrangement passing through a common point, and there is a translational component in the extrinsic parameters of the cameras, parallax may occur at the boundary portions of regions having different depths in the captured image. Parallax increases as the translational component between camera positions increases, and as the depth difference between regions in the image increases.

Due to inaccurate homography estimation, conventional tools for producing panoramic images have poor stitching performance for plain background images or noisy night images. The poor performance is because the number of extracted feature points is small, and these are not consistent in the temporal or spatial directions [7,10].

In real-world video applications such as sports and surveillance, multiple static cameras capture target scenes mainly composed of a ground, a far distant background, and foreground objects. In videos, foreground objects, especially dynamic objects, can draw more visual attention than the background. Typically, single or multiple homography-based stitching can be applied to such video sequences. If the estimated homographies are accurate, the stitched ground and background regions that make up most of the scenes and can be assumed to plane would not have parallax distortion. However, foreground objects at different distances from the cameras have depth differences from adjacent areas, leading to significant parallax distortion severely degrading the visual quality.

In this study, we focused on stitching video sequence pairs captured using static cameras in sports stadiums with running tracks enclosing a grass field. There are two challenges for stitching such video sequences. First, dynamic foreground objects in overlapped regions typically have parallax distortion. Second, the ground plane consisting of a grass field and a running track may not provide a sufficient number of feature points for homography estimation. This may cause misalignment distortion in the stitched ground region.

We propose a semantic segmentation-based video stitching method to reduce parallax and misalignment distortion in stitched video sequences. First, to reduce parallax distortion, video frames are divided into segments of different classes using a semantic segmentation module trained for sports stadium scenes. Stitching for matched segment pairs is performed assuming that these segments exist on the same plane. Second, to reduce the misalignment distortion for plain or noisy video frames, the homography is estimated by searching for consistent feature points in the temporal direction. Finally, the final video frames are stitched by stacking the stitched segments and foreground segments on the reference frame by descending order of area.

The contribution of the proposed method is three-fold. First, the proposed approach presents a reference framework for stitching videos, including foreground objects captured in specific scene environments such as sports stadiums. Although image or video stitching methods after foreground and background separation have been tried [11,14], parallax distortion still exists for static foreground regions [14] and multiple foreground regions [11]. A pre-trained semantic segmentation module can separate video frames into planar or multiple foreground segments of different classes and make per-segment stitching and stacking possible. For video stitching of other scene types, the semantic segmentation module should be re-trained with images from the scenes. Second, the proposed method can reduce the quality degradation of stitched videos by reducing the parallax distortion around foreground objects drawing great visual attention. Third, the proposed method can reduce the misalignment distortion in planar segments with simple texture or noise, such as the grass field in a typical sports stadium.

This paper is organized as follows: In Section 2, existing studies related to the reduction of parallax distortion in image or video stitching are explained. In Section 3, a semantic segmentation-based video stitching method is proposed for the reduction of parallax and misalignment distortion. In Section 4, the performance and effectiveness of the proposed method are evaluated. Finally, in Section 5, conclusions and future works are presented.

## 2. Related Works

Conventional stitching techniques to reduce parallax distortion can be divided into two groups. The first group selects a stitching seam between images and expresses only one piece of image information to the left and right of the seam to hide parallax and misalignment distortion [8,9,15,16,17,18]. The second group divides the image into small regions and then warps them locally by estimating the homography for each region [10,11,12,14,19,20].

### 2.1. Seam-Based Stitching

Yoon et al. estimated an initial homography between the first frames of a video sequence pair for stitching [15]. The homography for the following frame pair is calculated using the recent homography and the movement component of each camera. For the reduction of misalignment distortion, the homography is improved through block matching around feature points warped to the stitching frame plane. However, the accuracy of the homography for the following frames is affected by that of the initial homography. Additionally, when pixels in regions have different depths, misalignment distortion may occur due to inaccurate homography.

Jiang et al. warped an entire video frame using global homography and then divided the frame into a grid mesh and performed local warping to minimize the spatio-temporal cost function. Then, by selecting a seam to avoid moving objects, they reduced parallax distortion caused by the depth difference between foreground and background in the image. However, if there are few feature points extracted from foreground objects or if foreground objects show static movement, distortion occurs around the seam [8].

Kim et al. performed content-preserving warping by optimizing an energy function that sets the seam close to the midline of the overlapped region between frames for stitching, avoiding spatial misalignment and structures with sharp edges. To reduce the quality degradation of the stitched video caused by frequent changes of the seam during warping, they proposed a method that partially updates the seams around objects. However, since warping using a single homography does not consider the depth of each region in the video frame, misalignment or ghosting distortion may occur around the seam, except in object regions [16].

Seam-based stitching methods have in common that they define energy functions according to the researchers’ intention and establish seams for minimum energy [8,15,16]. However, these methods hide rather than reduce the misalignment distortion and thus are not a fundamental solution to reduce distortion. In our previous works, to reduce the misalignment distortion for scenes with plain textures or few feature points, we estimated homography using feature points extracted during a predetermined time interval [21,22]. We showed experimentally that these methods could reduce misalignment distortion in overlapped regions in video sequences without foreground objects [21,22].

### 2.2. Multiple Homography-Based Stitching

Gao et al. divided an image into a ground plane and a far-field background plane and estimated the homography of each plane. For each pixel, the authors took the distance to the nearest feature point as a weight for each plane and warped each pixel to the target plane using a weighted homography [12]. Yoon et al. proposed a method to reduce the amount of computation in Gao’s dual homography method [19]. The weight was calculated using the Mahalanobis distance from the center of the matching feature points to each pixel. However, this method involves a large amount of weight calculation per pixel and suffers from parallax distortion if there are moving foreground objects or small regions of different depths in each plane [12,19].

Zaragoza et al. divided the input image into several cell regions and estimated the homography of each cell using a direct linear transform (DLT) and applying weights according to the distance between the center point of each cell and the matched feature point [10]. This method is more effective at reducing parallax distortion than are the dual homography estimation methods [12,19]. However, for increased cell sizes, the estimated homography becomes inaccurate because regions of different depths exist in the same cell. For decreased cell sizes, the estimated homography becomes accurate, but the computational load increases. This method is not efficient at removing parallax distortion if there is a significant depth difference across the boundaries of large foreground regions in the video.

Zhang et al. proposed a method of processing the background and foreground separately to remove ghosting distortion in the foreground region. Background with a wide field of view is stitched using background models. Foreground regions are segmented by the background subtraction method and warped to the target image planes. For matched foreground regions, a larger region is selected as the final foreground [14]. This method treats all static regions without movement as background, so ghosting distortion may still occur in static regions of different depths in the background.

Lee et al. segmented an input image using mean-shift. They classified the segments into background and foreground based on the ratio of inliers of feature points through the global homography transformation. They performed stitching by estimating homography for background and foreground separately. However, this method cannot be applied to images with multiple foreground regions or with a foreground region with an insufficient number of feature points [11].

These studies attempt to reduce parallax distortion by estimating region-specific common homography. However, there is a limit to the reduction of parallax distortion because there still exist pixels of different depths in cells or regions due to the low performance of background and foreground separation [10,11,12,14,19].

Another approach for matching a video sequence pair with large parallax is to use activity features of moving objects [20,23,24]. Activity features are defined as a temporal series of binary values at each pixel position indicating foreground objects’ existence. The corresponding pixels in a different view with the activity feature that are most similar to that of the pixels in the reference view are selected for matching. Although these methods are robust for arbitrary orientation, zoom level, and lighting conditions, they are limited to video sequence pairs with moving objects in the overlapped regions.

## 3. Semantic Segmentation-Based Static Video Stitching

**Figure 1:**
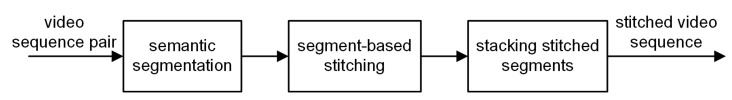
Proposed semantic segmentation-based video stitching.

In this section, a novel semantic segmentation-based static video stitching method is proposed to reduce parallax and misalignment distortion in stitched video frames. The proposed method, as shown in Figure 1, consists of three steps. In the first step, semantic segmentation is applied to an incoming video sequence pair. In the second step, for each background segment, multi-frame-based homography estimation and stitching are performed to generate stitched segments. Finally, all of the stitched segments and foreground segments are stacked on the reference view plane to generate panoramic video frames.

### 3.1. Semantic Segmentation and Matching

Most image and video stitching algorithms yield unconvincing results if an input image or video frame pair violates the following assumptions: (1) two images or video frame planes differ purely by rotation, or (2) the captured scene is effectively planar. However, in general image or video shooting environments, these assumptions are often violated, yielding parallax distortion in the stitching results. This parallax distortion is more severe in sports stadium videos with dynamic foreground objects of different depth from the surrounding areas than in landscape videos without foreground objects. Since dynamic foreground objects receive more visual attention than their surrounding regions, parallax distortion around them can significantly degrade the quality of stitched videos.

The purpose of the proposed algorithm is to reduce the parallax distortion around foreground objects and misalignment distortion in ground and background regions in stitching a video sequence pair captured using static cameras in sports stadiums. For semantic segmentation, the state-of-the-art algorithm DeepLab is employed [25]. DeepLab is trained to segment five classes of regions using images taken inside stadiums, including *ground*, *goalpost*, *building*, *human*, and *other*.

In the first step of the proposed algorithm, the reference and target frames are semantically segmented. Semantic labels are assigned to every pixel in the video frame by semantic segmentation. For scenes consisting of large planar segments, although pixels on the same segment may have different depths, they are assumed to be on the same plane. The pixels on small segments of foreground objects are also assumed to be on the same plane because the distance to the segment from the camera is a lot longer than the maximum difference of depth in the segment. The matched segment pairs, which have the same semantic class and are close to each other on the reference frame plane, are searched from the reference and target frames.

Let $F^{t}$ and $F^{r}$ denote the target and the reference video frames, respectively. After semantic segmentation, each video frame is divided into several disjoint segments. Each segment has its semantic class, such as *ground* ($R_{g}$), *building* ($R_{b}$), *human* ($R_{h}$), *goalpost* ($R_{p}$), and *other* ($R_{o}$). A background segment is defined as a static segment such as *ground*, *building*, *goalpost*, and *other*. The category of the foreground segment is defined into small semantic segments surrounded by background segments, i.e., a *human* semantic segment in our study.

For the *i*-th segment $R_{i}^{t}$ in the target frame and the *j*-th segment $R_{j}^{r}$ in the reference frame, an indicator function comparing the semantic classes for two segments is defined as follows:

(1) $$\begin{array}{c} I\left(R_{i}^{t},R_{j}^{r}\right)=\left\{\begin{array}{cc} 1, & T\left(R_{i}^{t}\right)=T\left(R_{j}^{r}\right)\\ 0, & \mathrm{otherwise} \end{array}\right., \end{array}$$

where T(·) represents the semantic class of the argument.

After matching the feature points extracted from these frames, a global homography, $H_{g}$, is estimated by random sample consensus (RANSAC), which relates a pixel p in $F^{t}$ to a pixel $p^{\prime }$ in $F^{r}$. Then, the segment $R_{i}^{t}$ in the target frame can be warped into the segment $R_{i}^{w}$ in the reference frame.

For each segment in the target frame, the matched segment in the reference frame is the one with a maximum overlapped area within a distance threshold. For the segment $R_{i}^{t}$, the index of the matched segment in the reference frame is found as follows:

(2) $$\begin{array}{c} I_{m}^{r}\left(i\right)=\left\{\begin{array}{cc} \underset{j}{arg min}\frac{\left|S\left(R_{i}^{w}\right)-S\left(R_{j}^{r}\right)\right|}{S(R_{j}^{r})}, & \forall j\;s.t.\;I\left(R_{i}^{t},R_{j}^{r}\right)=1\;\mathrm{and}\;d\left(R_{i}^{w},R_{j}^{r}\right)\leq d_{th}\\ \mathrm{null}, & \mathrm{otherwise} \end{array}\right., \end{array}$$

where $d(R_{i}^{w},R_{j}^{r})$, S(·), and $d_{th}$ represent the distance between the centroids of $R_{i}^{w}$ and $R_{j}^{r}$, the area of the argument, and the distance threshold, respectively.

### 3.2. Homography Estimation for Matched Segment Pairs

The correct alignment parameters need to be found between matched segment pairs while suppressing incorrect feature point extraction caused by time-varying noise. Primarily, segments of plain texture or small area may not provide a sufficient number of feature points for homography estimation by direct linear transformation [26].

**Figure 2:**
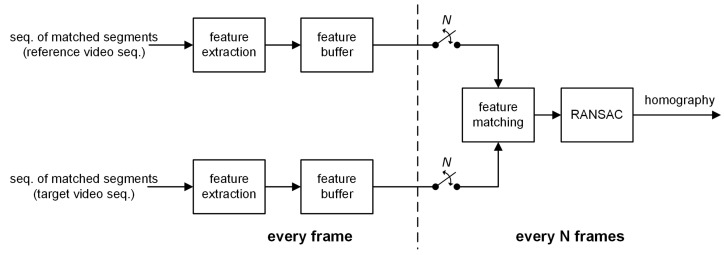
Homography estimation for a sequence of matched segment pairs.

In this section, a homography estimation method on a multi-frame basis is proposed to stitch matched segment pairs in static video camera environments. The proposed method is performed on every matched segment pair. For each side of the video sequences, as shown in Figure 2, feature points are extracted from the matched segment pair over an interval *N* using the SURF algorithm [27]. The extracted feature points are saved in the feature buffers until the end of the interval. Then, using the buffered feature points, a homography for the matched segment pair for the interval is estimated throughout feature matching and RANSAC [28].

In video stitching, there may exist time-varying noise and different illumination conditions. The proposed homography estimation method uses feature points extracted for multiple frame intervals. Feature points extracted multiple times at the same location can be considered as consistent against noise in the spatio-temporal domain. Multiple occurrences of feature points at the same location may increase their chances of being sampled by RANSAC in proportion to their number of occurrences [28]. Homography estimation is performed on background matched segment pairs, but not on foreground segments.

### 3.3. Panoramic Video Frame Synthesis Based on Segment-Based Stitching

In this section, a panoramic video frame synthesis method based on segment-based stitching is proposed. The left camera plane is selected as the reference view plane. After semantic segmentation and matching of segments, the segments can be grouped into three sets of segments. First, the sets $G_{b}^{t}$ and $G_{b}^{r}$ consist of matched pairs of background segments in the target and the reference frames, respectively. Second, the set $G_{f}^{r}$ consists of foreground segments or non-matched segments in the reference frame. Lastly, the set $G_{f}^{t}$ consists of non-matched segments in the target frame.

The target frame $F^{t}$ can be defined as the union of its segments, as follows:

(3) $$F^{t}=\overset{N^{t}}{\underset{i=1}{⋃}}R_{i}^{t},$$

where $N^{t}$ represents the number of segments.

Similarly, the reference frame $F^{r}$ is defined as follows:

(4) $$F^{r}=\overset{N^{r}}{\underset{i=1}{⋃}}R_{i}^{r},$$

where $N^{r}$ represents the number of segments.

The sets of matched background segments for the target and the reference frames are defined as follows:

(5) $$G_{b}^{t}=\left\{R_{i}^{t}|I_{m}^{r}\left(i\right)\neq \mathrm{null},1\leq i\leq N^{t}\right\}.$$

(6) $$G_{b}^{r}=\left\{R_{I_{m}^{r}\left(i\right)}^{r}|I_{m}^{r}\left(i\right)\neq \mathrm{null},1\leq i\leq N^{t}\right\}.$$

The set of the foreground or non-matched segments in the reference frame is defined as follows:

(7) $$G_{f}^{r}=F^{r}-G_{b}^{r}.$$

The set of non-matched segments in the target frame is defined as follows:

(8) $$G_{f}^{t}=F^{t}-G_{b}^{t}.$$

**Figure 3:**
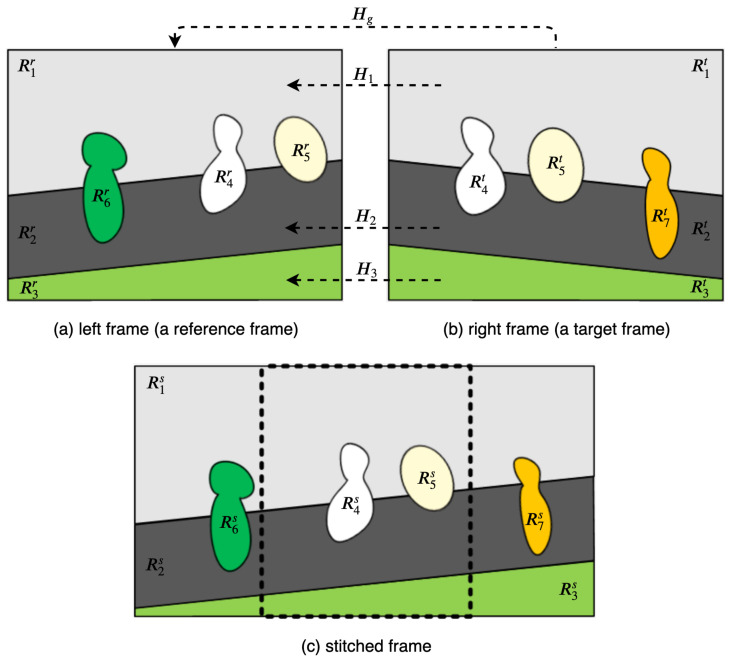
Segment-based homography estimation and stitching.

For the image pair shown in Figure 3, these sets can be constructed as $G_{b}^{t}=\left\{R_{1}^{t},R_{2}^{t},R_{3}^{t}\right\}$, $G_{b}^{r}=\left\{R_{1}^{r},R_{2}^{r},R_{3}^{r}\right\}$, $G_{f}^{t}=\left\{R_{7}^{t}\right\}$, and $G_{f}^{r}=\left\{R_{4}^{r},R_{5}^{r},R_{6}^{r}\right\}$.

For the warping and stitching of segments, sets of different semantic classes are handled in different ways. For each pair of matched background segments in the sets $G_{b}^{r}$ and $G_{b}^{t}$, a homography is estimated using the proposed multi-frame feature buffering during one interval. Then, the target background segment, $R_{i}^{t}$, is warped into the reference frame plane, aligned with its matched segment, $R_{I_{m}^{r}\left(i\right)}^{r}$. The stitched matched segment can be defined as follows:

(9) $$R_{i}^{s}=f\left(H_{i},R_{i}^{t}\right)⊕R_{I_{m}^{r}\left(i\right)}^{r},$$

where $f(H_{i},R_{i}^{t})$ and ⊕ represent the warping of the segment $R_{i}^{t}$ using the homography $H_{i}$ and the stitching operation of two segments, respectively.

In this paper, to show unwanted parallax distortion or misalignment distortion from inaccurate homography in the overlapped segment, if they exist, the warped target background segments and their matched segments are average blended into background stitched segments without post-processing, such as the seam-line selection, gain compensation, or multi-band blending used in [7,16,18].

Segments in the set $G_{f}^{r}$ are kept intact, regardless of the existence of matched segments. The matched foreground segments in the target frame are discarded without their warping to the reference frame plane. In Figure 3, $R_{4}^{r}$, $R_{5}^{r}$, and $R_{6}^{r}$ are kept intact in the reference frame plane.

Each segment in the set $G_{f}^{t}$ is warped to the reference frame plane using the homography estimated for the adjacent background segment having the highest number of edge pixels shared with the segment. In Figure 3, $R_{7}^{t}$ is warped to the reference frame plane using the homography $H_{2}$.

**Figure 4:**
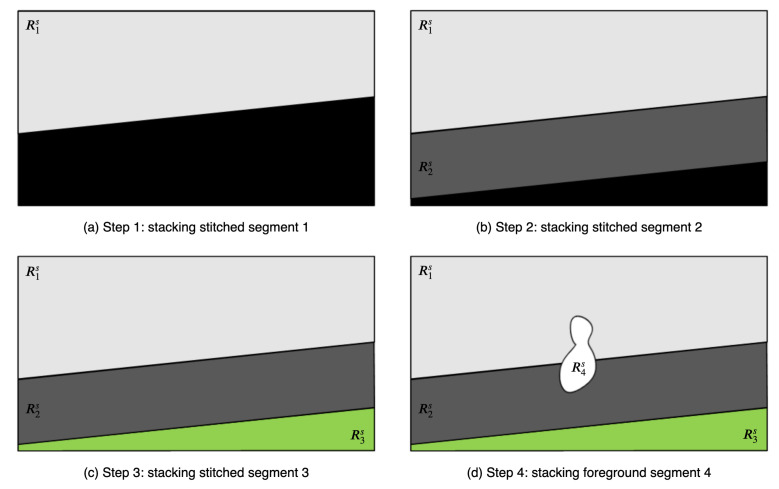
Panoramic frame synthesis.

If all processes of segment-based stitching and warping are performed, there are background stitched segments, warped non-matched segments from the target frame, and foreground or non-matched segments from the reference frame. These segments are stacked on the reference frame plane. First, background stitched segments are stacked according to the area of the segments, in descending order. Thereafter, foreground and non-matched segments are stacked irrespective of the order. In Figure 4, the stacking order of background stitched and foreground segments is shown. In this example, to synthesize the stitched video frame, the proposed algorithm stacks the segments in the order of $R_{1}^{s}$, $R_{2}^{s}$, $R_{3}^{s}$, and $R_{4}^{s}\left(=R_{4}^{r}\right)$. Then, the remaining unused segments are stacked as $R_{5}^{s}\left(=R_{5}^{r}\right)$, $R_{6}^{s}\left(=R_{6}^{r}\right)$, and $R_{7}^{s}$. The sequence of stacking foreground and non-matched segments is random.

## 4. Experimental Results

In this section, the experimental environments and results are presented for the proposed segment-based video stitching method.

### 4.1. Experimental Environments

**Figure 5:**
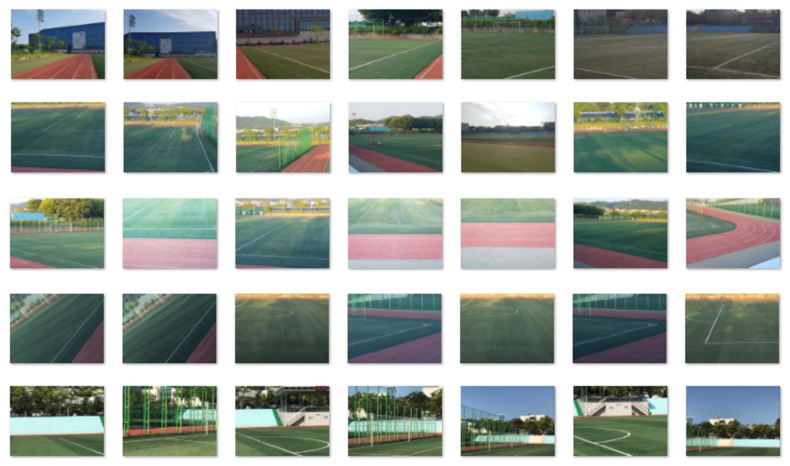
Image database for training of semantic segmentation module.

**Figure 6:**
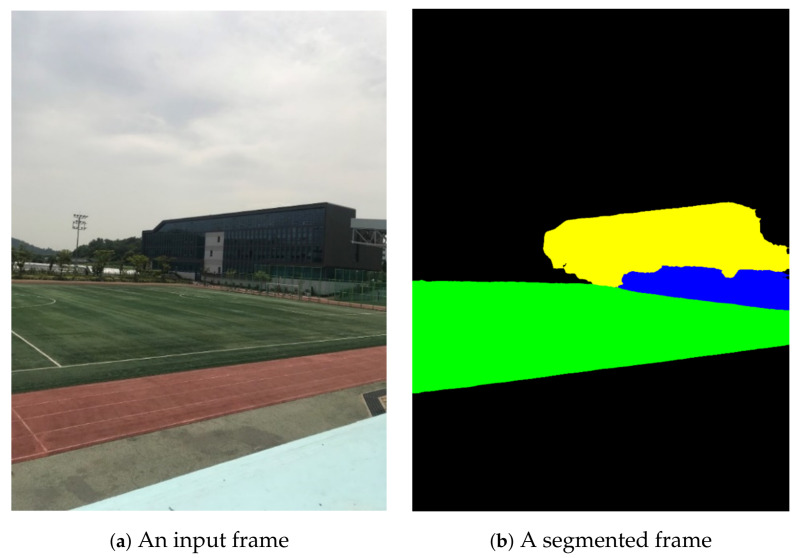
Example of semantic segmentation: green: *ground*; black: *other*; yellow: *building*; blue: *goalpost*.

The proposed video stitching method was implemented using the C++ programming language and OpenCV functions related to feature extraction, matching, and RANSAC operations [29]. The proposed method targets sports stadiums with running tracks enclosing a soccer field. For the training of the semantic segmentation tool, DeepLab, the training image dataset in Figure 5 was constructed; the dataset consists of 380 images captured at different locations with different viewing angles in a sports stadium located on a university campus. Five classes were defined for semantic segmentation: *ground*, *goalpost*, *building*, *human*, and *other*. Ground truths for semantic classes of segments were generated for every image for DeepLab training [25]. Figure 6 shows the results of semantic segmentation for test video sequences captured in the target sports stadium.

Four video sequence pairs, *seq1–4*, were shot at two sports stadiums for the performance evaluation of the proposed method. A static video camera pair installed with a separation of 1–2 m captured the inside of the stadiums from the stands. Each video sequence pair consists of two video sequences captured using the left and right video cameras. There exists a maximum 70% overlap in the field of view in the left and right video frames in each video sequence pair. The resolution and the frame rate of each video sequence are 1920×1080 and 30 Hz, respectively. For a quantitative performance evaluation of stitched video frames, a center video sequence was shot simultaneously with the video sequence pair *seq2*. The homography estimation interval *N* was set at 20 frames.

The quality of the video sequences stitched by the proposed method was compared with those stitched using the commercial software AutoStitch [7] and the cell-based homography estimation and stitching method APAP [10]. There are two methods of quality evaluation. The first method compares the misalignment and parallax distortion in the overlapped region. After Delaunay triangulation [30], the second method evaluates the geometric and pixel distortion of stitched or warped images based on an objective assessment method [31]. This method uses the left, center, and right video frames of the same scenes at different viewing angles. The panoramic image synthesized using the left and right video frames is aligned with the center image to compare geometric distortion and pixel distortion. Because the proposed method attempts to improve the alignment performance by reducing parallax distortion, the seam-line selection method used in AutoStitch was not implemented. The quality of AutoStitch was evaluated for two options: with seam-line selection (AutoStitch_Seam) and without seam-line selection (AutoStitch_NoSeam).

### 4.2. Region-Based Stitching Results

**Figure 7:**
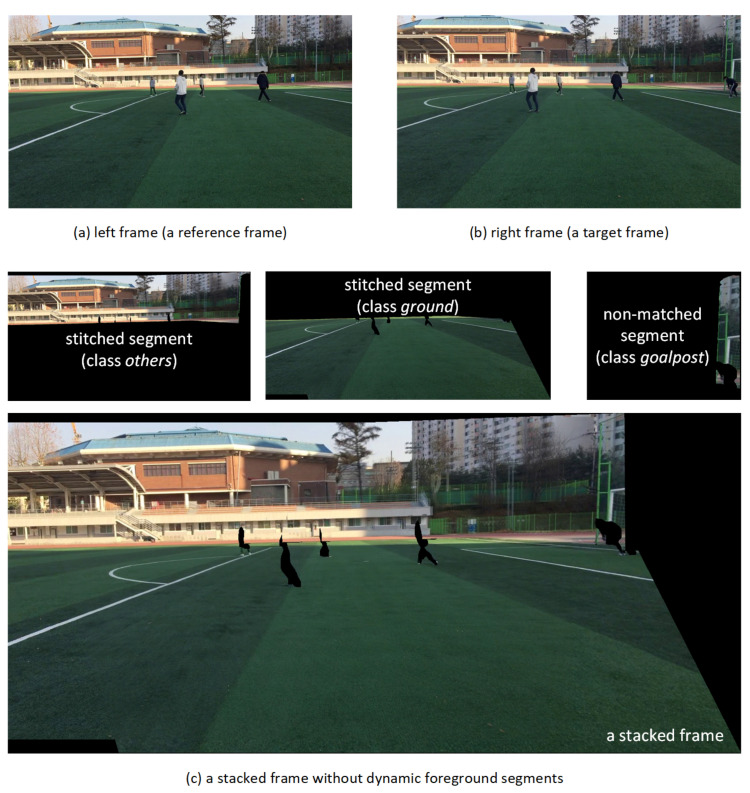
Segment-based stitching for background segments: *seq1*.

In this section, we present the stitched segments and their stacked panoramic video frames for each video sequence pair processed by the proposed method. As can be seen in Figure 7, the left frame was set as the reference plane for stitching, and the right frame was warped and aligned to the left frame. The left and right images of *seq1* overlap by 80%.

Each video frame is segmented into five semantic classes. In the target environment of this study, the foreground segments are human objects in the general sports stadium. For the foreground segments, it is difficult to estimate the homography consistently over a time interval because a sufficient number of feature points cannot be extracted. Thus, as described in Section 3.3, for each human object, the left foreground segment is selected without warping. For the other segments, the stitched segments are used for the composition of the final stitched frame.

**Figure 8:**
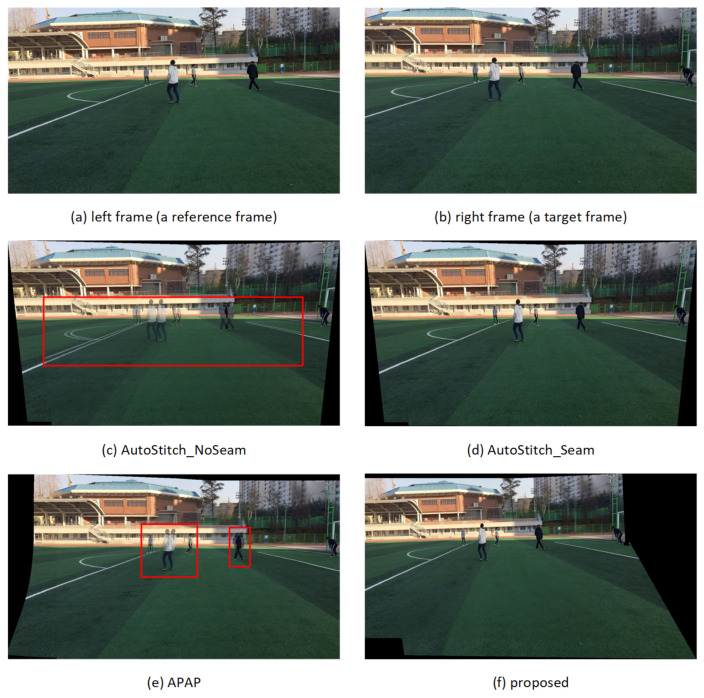
Quality comparison of stitched video frames: *seq1*.

Excluding the foreground segments, the matched background segment pairs on the frame boundaries are stitched and stacked on the reference view plane by order of area. Figure 7c shows a stacking example of the stitched *ground* and *other* segments and the non-matched *goalpost* segment in the reference view plane. Since the *goalpost* segment has no matched segment in the left frame, it is stacked after being warped to the reference view plane using the homography estimated for the adjacent *other* segment. If a matched foreground *human* segment exists, the foreground segment on the reference frame plane is stacked as is, without warping. If there is a non-matched foreground segment of *human* class, the warping and stacking process is performed in the same manner as for the non-matched *goalpost* segment. After the proposed stitching and stacking of foreground and background segments, the panoramic video frame shown in Figure 8f is synthesized.

### 4.3. Evaluation of Subjective Quality of Stitched Videos

**Figure 9:**
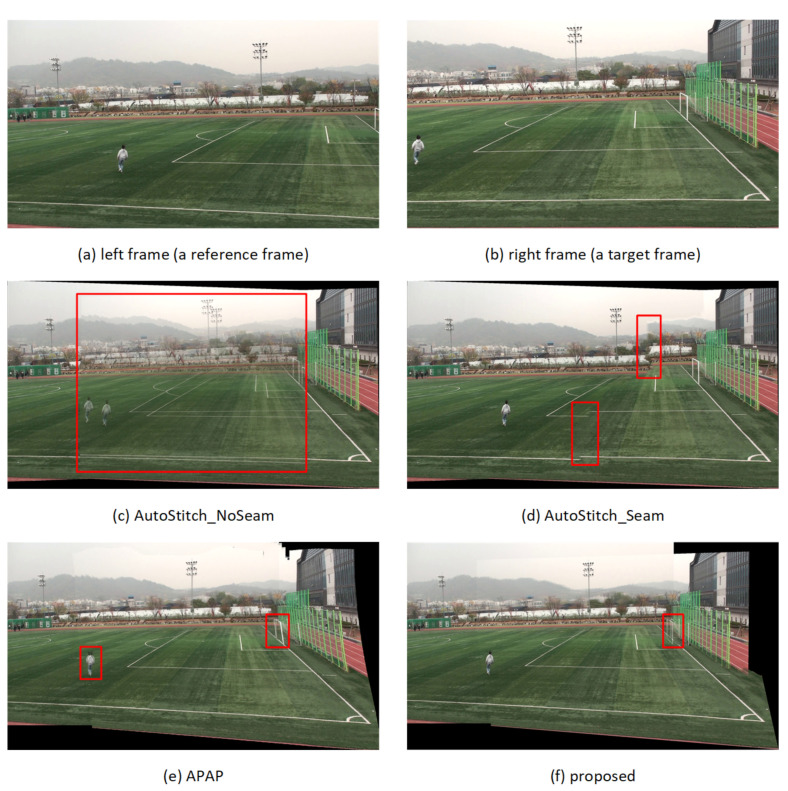
Quality comparison of stitched video frames: *seq2*.

**Figure 10:**
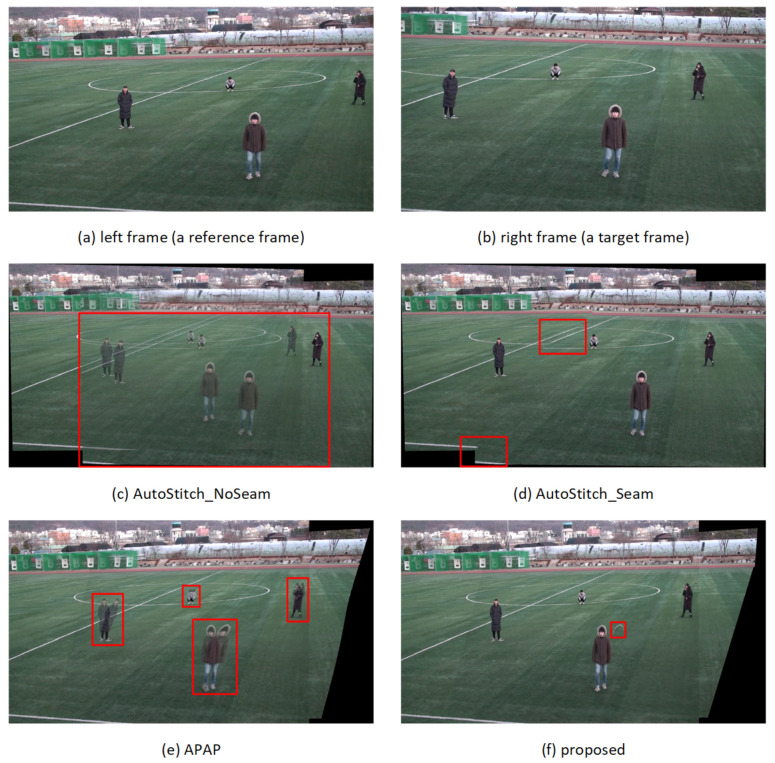
Quality comparison for stitched video frames: *seq3*.

**Figure 11:**
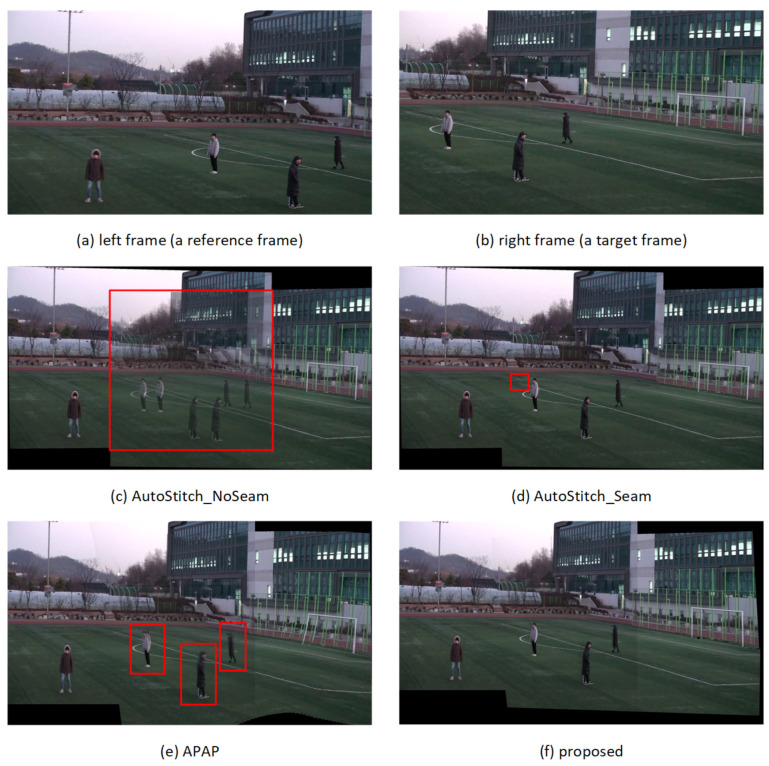
Quality comparison for stitched video frames: *seq4*.

The subjective quality of the stitched video sequences can be evaluated for misalignment distortion in *ground* and *other* segments and for ghosting distortion due to parallax in foreground segments. Figure 8, Figure 9, Figure 10 and Figure 11 show results of test video sequence pairs stitched by the proposed and existing methods.

The AutoStitch_NoSeam method warps the entire right frame using global homography and superimposes that frame on the reference frame. Global homography cannot be accurately estimated when the inter-frame relation does not exhibit pure rotation, or the segments have significant depth difference. Therefore, misalignment distortion and ghosting distortion of the foreground segment may occur in the entire frame. In Figure 8c, global homography is advantageously estimated for the *other* segment with a large number of feature points, but the results show significant misalignment distortion in the *ground* and *human* segments. In Figure 9c, Figure 10c and Figure 11c, it can be easily seen that misalignment distortion is substantial in all segments.

The AutoStitch_Seam method selects a seam showing little change between its left and right pixels and warps the pixels in the video frame selectively based on the seam. Therefore, no misalignment distortion of the foreground segment is noticeable. However, since a segment having a plain texture, such as the ground of the stadium, has no significant signal change in any direction, a wrong seam is likely to be set. In Figure 9d, Figure 10d and Figure 11d, there is misalignment distortion in the *ground* and *other* segments due to the incorrect seam. Only in *seq1* in Figure 8d is misalignment distortion not noticeable with the seam.

The APAP method can reduce the local misalignment distortion of *other* or *ground* segments without seam selection by dividing the input video frame into small rectangular cells and warping them using the homography estimated for each cell. However, if cells are set over several segments of significant depth difference, misalignment distortion still exists. In Figure 8e, Figure 9e, Figure 10e and Figure 11e, misalignment distortion is not noticeable in *ground* and *other* segments, but is noticeable in *human* and *goalpost* segments.

The proposed method stacks the foreground *human* segments in the overlapped region without warping, so ghosting distortion does not exist. Additionally, the proposed method shows no noticeable misalignment distortion because it stitches semantic segments using segment-based stitching and stacking and multi-frame-based homography estimation. In Figure 8f, Figure 9f, Figure 10f and Figure 11f, there is no misalignment distortion for any of the segments in *seq1–4*. However, if the boundary of a *human* segment in Figure 10f is not correctly segmented, there may, depending on the performance of the semantic segmentation, be an afterimage of the segment boundaries in the stitched video frame.

### 4.4. Objective Quality Evaluation of Stitched Videos

For objective quality evaluation of stitched videos, triangulation-based geometric distortion and pixel distortion measures were used [31]. For distortion calculation, the left and right video sequences for stitching and the central video sequence for comparison are required. Thirty video frames were taken from three video sequences (*seq2–4*). The stitched frames were warped to the central frame using the homography estimated by matching the feature points of the stitched and the central video frames.

Delaunay triangulation was performed using the matched feature points in the warped stitched and central frames [30]. Geometric distortion was used to calculate the average distance between matched feature points from the warped stitched and the central frames in the central frame plane. Pixel distortion calculates the PSNR between triangles on the warped stitched frame and their matched triangles on the central frame.

In the process of warping and aligning the stitched frame to the central frame plane, AutoStitch warps the entire frame using a global homography, as in the case of stitching the left and right video sequences. The APAP method divides the input video frames into small rectangular cells and warps them using the homography estimated for each cell. Since the proposed method performs segment-based stitching, the aligned stitched frame is synthesized by stacking the warped segments from the stitched frame.

Quantitative quality comparison results for the proposed, the APAP, and the AutoStitch methods are shown in Table 1. The segment or cell-based stitching methods, the proposed and the APAP methods, showed performance improvements in the geometric distortion over the frame-based stitching methods, AutoStitch_NoSeam and AutoStitch_Seam, especially for *seq2–3*. The proposed method showed performance improvements of around 2 dB in pixel distortion over other methods for *seq2–3*. Although all the methods showed similar geometric and pixel distortions for *seq4*, the proposed method shown in Figure 11 showed less parallax distortion and a smaller misalignment error in the overlapped region compared to other methods.

## 5. Conclusions

In this paper, we proposed a semantic segmentation-based video stitching method to reduce parallax and misalignment distortion between cameras. To eliminate parallax distortion, video frames are segmented into different semantic classes of segments. Assuming that a matched segment pair of the same semantic class exists on the same plane, segment-based stitching and stacking of the stitched segments are performed. To reduce misalignment in video sequences with simple texture or noise, homography is estimated using consistent feature points in the temporal direction. The proposed method outperformed existing methods for parallax, misalignment, geometric, and pixel distortions, especially in the plain *ground* segment and the foreground *human* segments that have significant depth differences from surrounding segments.

The proposed method has two limitations. First, if the boundary between neighboring segments is not accurately extracted, afterimages of the segment boundary remain in the adjacent region. Second, misalignment distortion may occur in background *other* segments in some video sequences due to the significant depth difference.

## Figures and Tables

**Table 1:** Objective quality evaluation.

Stitching Method	Geometric Distortion (Pixel)						Pixel Distortion (dB)		
	*seq2*		*seq3*		*seq4*		*seq2*	*seq3*	*seq4*
	*avg*	*std*	*avg*	*std*	*avg*	*std*			
AutoStitch_NoSeam	12.6	20.8	8.9	18.2	4.5	11.2	19.3	21.7	22.7
AutoStitch_Seam	11.7	15.5	10.3	16.9	4.8	11.5	21.1	21.9	**22.8**
APAP	8.5	**13.6**	**6.4**	11.7	4.9	9.8	21.0	22.0	22.3
Proposed	**6.6**	16.6	7.7	**10.8**	**4.1**	**8.9**	**23.8**	**24.2**	22.5

*avg* and *std* in column 2–7 represent the average and the standard deviation of the geometric distortion. The bold figure in each column is the best case.

## Data Availability

Not applicable.

## Abbreviations

The following abbreviations are used in this manuscript:

ICT	Information and Communications Technology
VR	Virtual Reality
UHD	Ultra High Definition
AR	Augmented Reality
FoV	Field of View
DLT	Direct Linear Transform
RANSAC	Random Sample Consensus
SURF	Speeded-Up Robust Features
